# The Effect of Disgust and Fear Modeling on Children’s Disgust and Fear for Animals

**DOI:** 10.1037/a0037228

**Published:** 2014-06-23

**Authors:** Chris Askew, Kübra Çakır, Liine Põldsam, Gemma Reynolds

**Affiliations:** 1 Department of Psychology, Kingston University

**Keywords:** anxiety, vicarious learning, fears, disgust, observational learning

## Abstract

Disgust is a protective emotion associated with certain types of animal fears. Given that a primary function of disgust is to protect against harm, increasing children’s disgust-related beliefs for animals may affect how threatening they think animals are and their avoidance of them. One way that children’s disgust beliefs for animals might change is via vicarious learning: by observing others responding to the animal with disgust. In Experiment 1, children (ages 7–10 years) were presented with images of novel animals together with adult faces expressing disgust. Children’s fear beliefs and avoidance preferences increased for these disgust-paired animals compared with unpaired control animals. Experiment 2 used the same procedure and compared disgust vicarious learning with vicarious learning with fear faces. Children’s fear beliefs and avoidance preferences for animals again increased as a result of disgust vicarious learning, and animals seen with disgust or fear faces were also rated more disgusting than control animals. The relationship between increased fear beliefs and avoidance preferences for animals was mediated by disgust for the animals. The experiments demonstrate that children can learn to believe that animals are disgusting and threatening after observing an adult responding with disgust toward them. The findings also suggest a bidirectional relationship between fear and disgust with fear-related vicarious learning leading to increased disgust for animals and disgust-related vicarious learning leading to increased fear and avoidance.

Disgust is considered to be one of the basic emotions and is protective, preventing contamination and ingestion of harmful substances (Rozin & Fallon, 1987). Like fear and anxiety, disgust is a defensive emotion (Davey, 2011) and is associated with certain fears and anxieties, in particular, blood-injection-injury phobias (de Jong & Merckelbach, 1998; Olatunji, Smits, Connolly, Willems, & Lohr, 2007), concerns with contamination in obsessive–compulsive disorder (e.g., Moretz & McKay, 2008; Muris et al., 2000), and fear of fear-relevant but nonthreatening animals (Davey, Forster, & Mayhew, 1993; Matchett & Davey, 1991; Ware, Jain, Burgess, & Davey, 1994). Fear and anxiety are both components of anxiety disorders but are distinct from one another (Davey, 2011): Anxiety is typically characterized as the anticipation of threat and experienced through feelings of fear, apprehension, and uncertainty, but fear is a direct response to a specific perceived threat. Disgust may be important for different fears and anxieties in different ways; for example, fear of blood can be predicted by both disgust propensity (the tendency for individuals to respond with disgust to disgust-eliciting stimuli) and disgust sensitivity (the tendency for individuals to experience disgust as particularly unpleasant), whereas disgust propensity is more important than disgust sensitivity in the prediction of spider fear (van Overveld, de Jong, Peters, Cavanagh, & Davey, 2006).

The relationship between disgust and spider fear appears to be particularly pronounced (Davey, 1994b; de Jong & Merckelbach, 1998; de Jong & Muris, 2002; Mulkens, de Jong, & Merckelbach, 1996; Olatunji, 2006; Thorpe & Salkovskis, 1998). The phobic response to spiders is typically made up of a combination of both fear and disgust (de Jong, Peters, & Vanderhallen, 2002; Sawchuk, Lohr, Tolin, Lee, & Kleinknecht, 2000; Tolin, Lohr, Sawchuk, & Lee, 1997), but disgust is a stronger predictor of spider avoidance than anxiety (Woody, McLean, & Klassen, 2005). Moreover, disgust responses to stimuli appear to show greater resistance to extinction than fear (Mason & Richardson, 2010; Olatunji, Forsyth, & Cherian, 2007), although they are not routinely targeted during treatment (de Jong & Muris, 2002). Disgust-evoking stimuli also appear to hold attention at earlier stages of visual processing than fear-evoking stimuli (van Hooff, Devue, Vieweg, & Theeuwes, 2013), supporting a role for information processing biases in disgust. An example of this is evidence of attentional bias for disgust-related words in a Stroop task following emotional priming; response latencies and memory for disgust words were also found to be positively associated with disgust sensitivity after disgust priming (Charash & McKay, 2002). Similar Stroop task biases are found in many psychopathologies (Williams, Mathews, & MacLeod, 1996), including spider phobia (e.g., Kindt & Brosschot, 1997), social anxiety, and panic disorder (Hope, Rapee, Heimberg, & Dombeck, 1990).

The disgust emotion does not appear to be present at birth, but develops at some time before the age of 5 years (Rozin & Fallon, 1987). One way that stimuli are believed to acquire disgust-eliciting status is via “contamination”: by coming into contact with something that is already disgusting (Fallon, Rozin, & Pliner, 1984). Evidence suggests that these disgust contamination responses do not develop until approximately 7 years of age, although an implicit understanding of contamination may be present much earlier (Brown & Harris, 2012; Brown, Harris, Bell, & Lines, 2012). The primary adaptive function of disgust may be to protect against harm (Rozin & Fallon, 1987). Matchett and Davey’s (1991) disease-avoidance model suggests that disgust plays such an important role in some fears because it leads to avoidance in an attempt to avert contamination. Many common animal fears may be the result of the animals first becoming either associated with the spread of disease, dirt or contamination, or possessing disgust-evoking perceptual features (e.g., looking like mucus or feces). Once this disgust status is acquired, cultural or familial learning processes can lead to fear development (Matchett & Davey, 1991). The association between a stimulus and disease or contamination may be historic, as is likely to be the case with rats or cockroaches, but may merely be superstitious or opportunistic, such as the association of spiders with contamination and disease in Europe in the Middle Ages (Davey, 1994a, 2011). Thus, threat- or disgust-related beliefs and associations, even if unfounded, may affect individuals’ fear and avoidance for stimuli.

The model suggests then that disgust is not only associated with anxiety but also implicated in the etiology of certain fears. Although associations between disgust and some stimuli are well established, correlational studies do not explain the mechanisms by which they occur. Experimental evidence is required to clarify these mechanisms. One way disgust might lead to fear may be by changing how threatening an individual thinks a stimulus is. Although inducing disgust does not in itself appear to directly increase levels of reported anxiety (Marzillier & Davey, 2004), it can lead to participants choosing more threatening interpretations of words (Davey, Bickerstaffe, & MacDonald, 2006). Inducing threat-interpretation bias has been shown to increase anxiety (Mathews & Mackintosh, 2000), so this suggests that one link between disgust and anxiety may be via such cognitive biases (Davey, 2011). Consistent with this, Webb and Davey (1992) found increases in participants’ fear of fear-relevant small animals after they watched a revulsive video (hospital procedures), suggesting that disgusted mood can facilitate fear of certain stimuli.

Rozin and Fallon (1987) argued that children likely learn that certain stimuli are disgusting via the verbal and nonverbal expressions of others. This has obvious parallels with Rachman’s (1977) suggestion that fears can be acquired via verbal information and vicarious learning. Studies suggest that animal fears develop early, typically beginning around 8.6 years of age on average (Öst & Treffers, 2001), with 62% first showing between 5 and 9 years of age (Öst, 1987); thus, it makes sense for experimental studies to look at the development of fear in children around this age. Field and colleagues have presented a wealth of evidence showing that verbal threat information about animals can increase fear responses to animals in children around this age, including increased fear beliefs, avoidance, and heart rate responses to animals (e.g., Field & Lawson, 2003; Field, Lawson, & Banerjee, 2008; Field & Schorah, 2007). In an adaptation of Field et al.’s experimental paradigm, Muris, Mayer, Huijding, and Konings (2008) gave 9- to 13-year-olds disgust- and cleanliness-related information about animals. Results indicated that both disgust beliefs and fear beliefs increased for animals about which children had received disgust-related information and decreases were found for cleanliness-related information. Follow-up studies also showed similar effects when disgust was nonverbally induced (Muris, Huijding, Mayer, & de Vries, 2012) and a bidirectional relationship between disgust and fear in which disgust-related information increased fear beliefs and threat information increased disgust (Muris et al., 2009).

Rozin and Fallon (1987) suggested that stimuli might become associated with disgust via social referencing: Children might learn that an animal or object is disgusting after observing someone else acting disgusted in response to it. There is evidence that fear can be learned in this way; for example, young infants (15–20 months of age) showed increased fear and avoidance of a toy snake and spider after their mothers indicated that they were scary and unpleasant (Gerull & Rapee, 2002), and this has also been observed for flower and mushroom stimuli (Dubi, Rapee, Emerton, & Schniering, 2008). Similar effects have been demonstrated in older children (7- to 9-year-olds), who showed increased fear beliefs and avoidance for novel animals after seeing pictures of them together with pictures of fear faces (Askew & Field, 2007; Askew, Kessock-Philip, & Field, 2008). Likewise, children may also be able to learn that stimuli are disgusting via observation of others. Each child in Muris, Mayer, Borth, and Vos’s (2013) study watched as the experimenter showed their mother boxes of specimens conveying information about attributes of the eating, sleeping, and grooming habits of previously unknown animals. Specimens were designed to either cause revulsion in parents toward the animal (e.g., worms as food) or be neutral (e.g., fruit as food). Children’s feelings of disgust for the animals was found to be affected by verbal information from parents, but there was no effect of the mother’s nonverbal expressions (e.g., facial expressions and gestures) on children’s disgust or fear feelings for animals. A possible explanation for the latter finding could be that the disgust manipulation, although high in ecological validity, may have been relatively mild (or at least inconsistent across participants) in terms of disgust conveyed nonverbally by parents to children.

One way to avoid this would be for the experimenter to maintain control over the intensity of the information conveyed to children during vicarious learning. For example, Askew and Field (2007) presented children (7–9 years of age) with pictures of novel Australian marsupials (the quoll, quokka, and cuscus) together with emotional faces in a series of animal–face pairing trials: one animal was seen with fear faces (fear-paired), one with happy faces (happy-paired), and one with no faces (control). Children’s fear beliefs for fear-paired animals increased and were still indirectly detected 3 months later. Children also avoided these animals more than control animals in a behavioral task. Further studies using the same procedure indicated that vicarious fear learning occurs for a range of stimuli, including flowers and snakes (Askew, Dunne, Özdil, Reynolds, & Field, 2013), and is similar whether the model is the child’s mother or a complete stranger (Dunne & Askew, 2013). Askew and Field (2007, 2008) have argued that stimulus–stimulus associative learning processes underpin the vicarious fear learning procedure such that the animal or object acts as the conditioned stimulus (CS) and becomes associated with the fear-related responses of the model acting as an unconditioned stimulus (US). This methodology could also be useful to investigate the effect of disgust vicarious learning on children’s disgust and fear for animals.

The current study adapted Askew and Field’s (2007) methodology to show children pictures of novel Australian marsupials (CSs) together with faces expressing disgust (USs). Children’s disgust beliefs were measured before and after vicarious learning to determine whether there was any effect of pairing animals with disgust faces. It was expected that the disgusted responses would become associated with the animals and lead to increases in children’s disgust-related beliefs for them. Given the strong causative links in the literature between disgust and fear, measures of children’s fear beliefs and avoidance preferences for the animals were also taken. It was predicted that disgust vicarious learning would lead to increases in children’s fear beliefs and avoidance for the animals.

## Experiment 1

### Method

An adaptation of Askew and Field’s (2007) vicarious learning procedure was used. Children saw each animal CS presented together with either disgust face USs (disgust-paired), happy face USs (happy-paired), or alone (unpaired control condition) in a within-subjects design. This was counterbalanced across children, so that there were three counterbalancing groups in which each animal was paired with each face type or was unpaired. Self-reported fear beliefs and disgust beliefs for animals were measured before and after vicarious learning using a version of the Fear Beliefs Questionnaire (FBQ; Field & Lawson, 2003). In addition, children’s avoidance preferences were measured using a nature reserve task (NRT; Field & Storksen-Coulson, 2007).

#### Participants

Sixty-two children between the ages 7 and 10 years (*M* = 110.66 months, *SD* = 10.05) were recruited from a primary school in Surrey, United Kingdom (30 boys, 30 girls) between the ages 7 and 10 years (*M* = 110.66 months, *SD* = 10.05). Headteachers were informed about the procedures and gave consent for the study to take place at the school. Parents/caregivers were fully informed about what their child would be asked to do via a letter sent home from the school that the child had to return signed by parents. Informed opt-in consent was obtained from parents/caregivers and all children gave verbal assent. There were no children with consent who did not also give verbal assent. No children were excluded for any reason at the recruitment stage of the study. However, two children were excluded from all analyses because of failure to follow instructions and data from a further four children’s Disgust Beliefs Questionnaires (DBQs) could not be included in analyses because of unanswered questions. Details about the socioeconomic status or ethnicity of the children were not recorded.

#### Materials

##### Stimuli

Animal stimuli (CSs) consisted of three different pictures (400 × 400 pixels) of three Australian marsupials (quokkas, quolls, and cuscuses), nine pictures in total. These animals were chosen because U.K. children are unlikely to know them (e.g., Dunne & Askew, 2013) or have prior fear or disgust beliefs for them. They have been successfully used in many similar vicarious learning procedures (e.g., Askew & Field, 2007; Askew et al., 2008; Dunne & Askew, 2013). Emotional face stimuli (USs) consisted of 10 portrait pictures (400 × 485 pixels) of two facial expressions (20 pictures in total) taken from the NimStim Set of Facial Expressions (Tottenham, Borscheid, Ellertsen, Marcus, & Nelson, 2002). Ten of the faces depicted disgust (five men, five women) and 10 depicted happiness (five men, five women).

##### FBQ

A computerized version of the FBQ (Field & Lawson, 2003) was used to measure fear beliefs for the animals before and after vicarious learning. This consisted of seven questions for each animal, such as “Would you be happy to have a quokka/quoll/cuscus for a pet?” Four questions were reverse scored. Children responded on a 5-point scale (0 = *no, not at all*; 1 = *no, not really*; 2 = *don’t know/neither*; 3 = *yes, probably*; 4 = *yes, definitely*). The final score was divided by the number of questions to produce a score ranging from 0 to 4 for each animal in which 0 was the lowest and 4 the highest level of fear beliefs. Internal consistency (Cronbach’s alpha) was moderately high before learning for the three animals: α = .68 for the cuscus subscale; α = .69 for the quokka subscale; α = .65 for the quoll subscale; and high after learning for the three animals: .84, .80, and .84, respectively.

##### DBQ

A DBQ was completed by children to measure their disgust attitudes for the animals before and after vicarious learning. This was almost identical to the Muris et al. (2008) scale with some small modifications to wording. It consisted of three questions: “Would you carefully wash your hands if you had touched a quokka/quoll/cuscus?” “Would you hold your nose if you were close to a quokka/quoll/cuscus?” “Would you prefer to wear gloves if you had to stroke a quokka/quoll/cuscus?” Responses were given on the same scale as the FBQ and were also averaged to give a score ranging from 0 (lowest) to 4 (highest). Internal consistency for the three animal scales was relatively low before learning: α =.54 for the cuscus subscale, α = .60 for the quokka subscale, and α = .71 for the quoll subscale; and was similar for the three animals after learning: .66, .66, and .58, respectively. The relatively low reliability scores were not that surprising because the DBQ consists of only three items and Cronbach’s alpha is often lower for smaller scales. To test this, we combined the quokka, cuscus, and quoll subscales into a single general “disgust for unfamiliar animals” scale. Before learning, Cronbach’s alpha for this scale was .85, and after learning, it was .79, confirming that small scale size may have been responsible for the low internal consistency of the animal subscales.

##### NRT

The NRT (Field & Storksen-Coulson, 2007) was used to measure children’s avoidance preferences for disgust-paired and unpaired animals after vicarious learning. The nature reserve itself consisted of a green-painted wooden board (450 mm × 600 mm), embellished with fabric flowers and plastic trees positioned around the edge so that children were unable to “hide behind” them. Children were told the animals live in the nature reserve; over two trials, the disgust-paired and the unpaired animals were placed, one at a time, at one end of the board. Children were then asked to place a Duplo figure representing themselves on the board where they would prefer to stand if visiting the reserve; the distance (in millimeters) from the center of the animal picture to the Duplo figure was measured to determine children’s avoidance preferences: The farther children placed the figure from the animal, the higher the level of preferred avoidance.

#### Procedure

The experiment was automated on a Pentium 4 laptop computer with a 15-in. monitor, using software custom written (Field, 2010) in Visual Basic.net with ExacTicks 1.10 (Ryle Design, 1997). Pictures of each of the three marsupials (40 × 40 mm) were shown to children and they completed the DBQ. Next, children completed the FBQ: Each question was presented at the top of the screen above a picture of the respective animal and children used a mouse to click an on-screen button displaying their response. Following the questionnaires, the vicarious learning phase commenced with a picture of an animal appearing on the screen for 1 s, followed by a picture of either a disgusted, happy, or no face appearing next to the animal on the opposite side of the screen for a further 1 s; each pairing trial lasted 2 s in total. Timings were identical to previous, similar studies (e.g., Askew & Field, 2007; Askew et al., 2008, 2013; Dunne & Askew, 2013). The order of animal images and pairing type (disgust-paired, happy-paired, or unpaired) was random. Each child was presented with one animal (e.g., a quokka) with 10 faces (e.g., disgust), a second animal (e.g., a quoll) with 10 faces (e.g., happy), and the third animal (e.g., a cuscus) appeared alone with no faces (unpaired) 10 times. The condition in which each animal was presented was counterbalanced across children. Following vicarious learning, the DBQ and FBQ were administered a second time, followed by the NRT. Children were debriefed and given age-appropriate information and worksheets about the animals.

### Results

#### Fear beliefs

**Figure 1 fig1:**
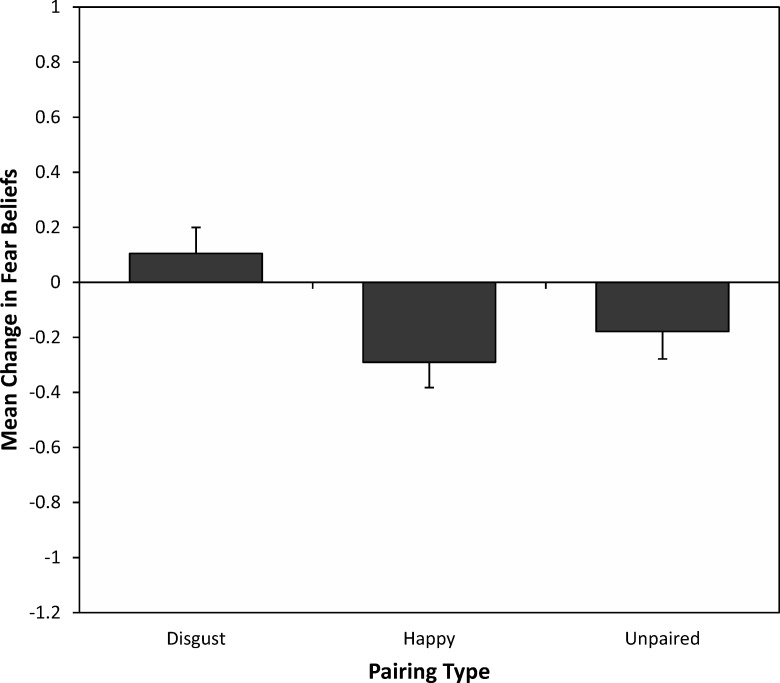
Mean (and *SE*) changes in self-reported fear beliefs for happy-paired, disgust-paired, and unpaired animals.

Mean changes in self-reported fear beliefs for each pairing type (positive, disgust, and unpaired) are displayed in Figure 1. An initial three-way 3 (pairing type: disgust, happy, unpaired) × 2 (time: before vs. after) × 3 (counterbalancing order: cuscus disgust-paired, quokka disgust-paired, and quoll disgust-paired) mixed analysis of variance (ANOVA) was conducted to check that learning was not greater in any of the counterbalancing groups. There were no significant main effect or interactions involving counterbalancing group and therefore no indication that learning was influenced by the visual appearance of any specific animal. Thus, the analysis was collapsed into a two-way 3 (pairing type: disgust, happy, unpaired) × 2 (time: before vs. after) mixed ANOVA to test predictions that disgust vicarious learning would increase fear beliefs. There was a borderline significant main effect of time, *F*(1, 59) = 3.89, *p* = .053, η_p_^2^ = .062, and significant main effect of pairing type, *F*(2, 118) = 6.19, *p* = .003, η_p_^2^ = .095. The crucial Pairing Type × Time interaction was significant, *F*(2, 118) = 5.17, *p* = .007, η_p_^2^ = .081, indicating an effect of vicarious learning on children’s fear beliefs for the animals. Planned contrasts did not find significant changes in fear beliefs for happy-paired animals (prelearning: *M* = 1.80, *SD* = 0.70; postlearning: *M* = 1.51, *SD* = 0.88) compared to unpaired animals (prelearning: *M* = 1.86, *SD* = 0.69; postlearning: *M* = 1.68, *SD* = 0.87), *F*(1, 59) = 1.04, *p* = .31, *r* = .13. However, there was a significant increase in fear beliefs for disgust-paired animals (prelearning: *M* = 1.85, *SD* = 0.66; postlearning: *M* = 1.96, *SD* = 0.75) compared to unpaired animals, *F*(1, 59) = 4.41, *p* = .040, *r* = .26, indicating that seeing animals with faces expressing disgust increased children’s fear beliefs for animals.[Fig-anchor fig1]

#### Disgust

**Figure 2 fig2:**
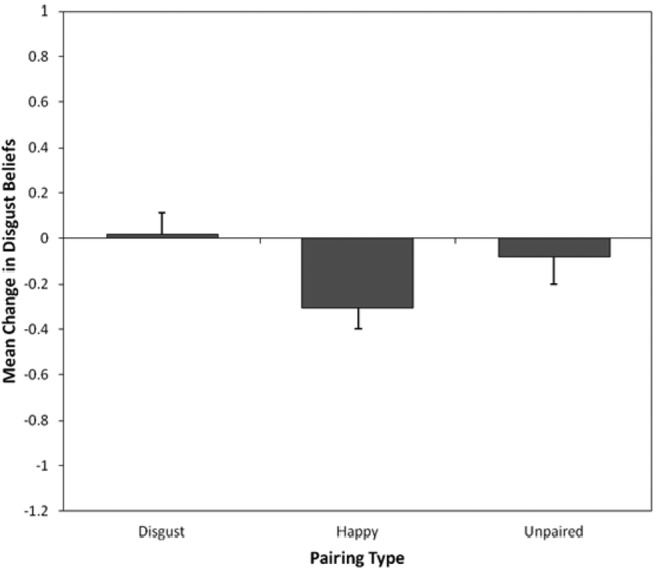
Mean (and *SE*) changes in self-reported disgust beliefs for happy-paired, disgust-paired, and unpaired animals.

Figure 2 displays mean changes in self-reported disgust ratings for each pairing type. An initial three-way 3 (pairing type: disgust, happy, unpaired) × 2 (time: before vs. after) × 3 (counterbalancing order: cuscus disgust-paired, quokka disgust-paired, and quoll disgust-paired) mixed ANOVA conducted on disgust beliefs found no significant main effects or interactions involving counterbalancing order. Therefore, the analysis was collapsed into a two-way 3 (pairing type: disgust, happy, unpaired) × 2 (time: before vs. after) mixed ANOVA. There was no significant main effect of time, *F*(1, 55) = 3.06, *p* = .086, η_p_^2^ = .05, but there was a significant effect of pairing type, *F*(2, 110) = 4.25, *p* = .017, η_p_^2^ = .07. The important Pairing Type × Time interaction was significant, *F*(2, 110) = 3.37, *p* = .038, η_p_^2^ = .06, indicating an effect of vicarious learning on children’s disgust beliefs. Follow-up comparisons found no significant difference in changes in disgust beliefs for disgust-paired animals (prelearning: *M* = 2.42, *SD* = 0.84; postlearning: *M* = 2.44, *SD* = 0.92) compared to unpaired animals (prelearning: *M* = 2.30, *SD* = 0.80; postlearning: *M* = 2.21, *SD* = 0.90), *F*(1, 55) = 0.67, *p* = .42, *r* = .11, or happy-paired animals (prelearning: *M* = 2.39, *SD* = 0.88; postlearning: *M* = 2.09, *SD* = 0.83) compared to unpaired animals, *F*(1, 55) = 3.08, *p* = .085, *r* = .23.[Fig-anchor fig2]

#### Avoidance preferences

On average, children placed themselves farther away from disgust-paired animals (*M* = 19.48 cm, *SD* = 12.35) than unpaired animals (*M* = 16.65 cm, *SD* = 12.04). A paired-samples *t* test (one-tailed) indicated, as predicted, that children preferred to avoid the disgust-paired animal compared to the unpaired animal, *t*(59) = 1.72, *p* = .045, *r* = .22.

#### Relationship between fear beliefs, disgust beliefs, and avoidance preferences

Controlled change scores for fear beliefs, disgust beliefs, and avoidance preferences were calculated for disgust-paired animals. These were mean change scores for disgust-paired animals relative to changes for the unpaired control animals and were simply calculated as the difference between mean changes for disgust-paired animals and mean changes for unpaired animals. For example, children’s controlled changes in fear beliefs were computed by subtracting the mean change in fear beliefs for unpaired animals from the mean change in fear beliefs for disgust-paired animals. There were significant correlations between controlled increases in fear beliefs and disgust beliefs, *r*(54) = .42, *p* = .001, and controlled increases in fear beliefs and avoidance preferences, *r*(58) = .47, *p* < .001. Similarly, controlled increases in disgust beliefs correlated with controlled avoidance preferences for disgust-paired animals, *r*(54) = .53, *p* < .001. This demonstrated that increases in children’s fear beliefs and avoidance preferences following disgust-related vicarious learning were directly related to increases in their disgust beliefs.

**Figure 3 fig3:**
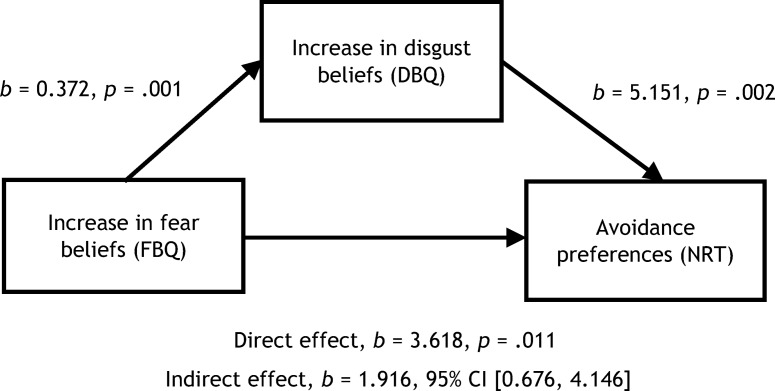
Mediation model of increases in children’s fear beliefs following disgust vicarious learning as a predictor of avoidance preferences and mediated by disgust beliefs for the animals. DBQ = disgust beliefs questionnaire; FBQ = fear beliefs questionnaire; NRT = nature reserve task; CI = confidence interval.

Avoidance preferences are likely to be a consequence of increased fear beliefs for animals. One possibility is that disgust beliefs play a role in the relationship between fear beliefs and avoidance, so that the effect of increasing fear beliefs on avoidance is greater when disgust beliefs for an animal are also high. If this is the case, we would expect disgust beliefs to mediate the effect of fear beliefs on children’s avoidance preferences for animals following vicarious learning. A Hayes-style (2013) mediation analysis confirmed this: There was a significant indirect relationship between increases in fear beliefs and children’s avoidance preferences through increases in disgust beliefs (using controlled change scores again), *b* = 1.916, bootstrapped confidence interval (BCa CI) [0.676, 4.146], with effect size *k*^2^ = .174, BCa CI [.065, .298]. Figure 3 shows the model for this mediation effect.[Fig-anchor fig3]

## Experiment 2

Experiment 1 showed that children’s fear beliefs for animals increase as a result of seeing them together with pictures of people looking disgusted and these increases are associated with increases in disgust beliefs for the animals. In addition, children preferred to avoid animals they had seen with disgust faces. This avoidance was predicted by increases in fear beliefs for the animals and mediated by increases in disgust beliefs. However, disgust beliefs for animals did not increase significantly for animals following disgust vicarious learning. Unfortunately, this may have been due, in part, to the low reliability of the measure used. Thus, it was not possible to determine conclusively from Experiment 1 whether disgust modeling led directly and independently to increased fear beliefs or whether these changes were in parallel with increased disgust beliefs.

There may be differences between the effects of fear and disgust vicarious learning; for example, fear vicarious learning may have a greater effect on fear beliefs than disgust vicarious learning. Conversely, disgust vicarious learning may lead to greater increases in learned disgust than fear vicarious learning. Given the role of disgust in fears, another possibility is that fear vicarious learning leads to increases in disgust beliefs, as well as having established effects on fear beliefs and avoidance (e.g., Askew & Field, 2007; Askew et al., 2013). Feelings of disgust may play a part in animal fears even when the original learning was fear-related rather than disgust-related. If fear vicarious learning does increase disgust, and disgust is an important element in some fears, avoidance preferences may be mediated by these increases in disgust, just as they are in disgust vicarious learning.

To examine this in more detail, Experiment 2 replicated the first experiment with two additions. First, an additional measure of disgust beliefs for animals was created because the reliability of the scale used in Experiment 1 was unclear. Second, in addition to a group of children who saw disgust vicarious learning, a second group saw fear faces during vicarious learning. This meant that the effect of fear vicarious learning on disgust beliefs could also be examined and comparisons could be made between the effects of fear vicarious learning and disgust vicarious learning on children’s fear beliefs. Given the overlap between fear and disgust and the fact that they are both negative emotions and facial expressions, the two types of learning were manipulated separately in a between-groups design. This design meant that there was no chance of contamination of fear- or disgust-related responses from either the fear- or disgust-paired animal to the other paired animal, while still allowing for the two types of learning to be compared. Given the findings from Experiment 1 and previous fear vicarious learning studies (e.g., Askew & Field, 2007), it was expected that disgust vicarious learning would increase fear beliefs and avoidance preferences for animals, and fear vicarious learning would increase fear beliefs and avoidance preferences. Again, given the overlap between fear and disgust in animal fears, corresponding increases in the additional measure of disgust were also expected and these were predicted to be associated with increases in fear measures.

### Method

The manipulation in Experiment 2 was essentially the same as that in Experiment 1; however, this time there were two groups of children. Approximately half of the children were presented with an identical vicarious learning procedure as that in the previous experiment: One animal was seen with disgust faces, one with happy faces, and one with no faces. The remaining children saw one animal with fear faces (fear-paired), one with happy faces (happy-paired), and one with no faces (unpaired), also in a within-subjects design. Thus, the vicarious learning procedure was identical for this group except that children always saw one of the animals with fear faces rather than disgust faces. Again, the type of face seen with a specific animal was counterbalanced across children. Self-reported fear beliefs and two measures of disgust beliefs for animals were compared before and after vicarious learning along with avoidance preferences again.

#### Participants

Fifty-eight children (25 boys, 33 girls) were recruited from three schools across the United Kingdom; they were between the ages of 7 and 10 years (*M* = 109 months, *SD* = 8.75). Twenty-nine children were randomly allocated to the disgust vicarious learning group, and 29 children were randomly allocated to the fear vicarious learning group. As in Experiment 1, informed consent was obtained from parents/caregivers and children gave verbal assent. No child who had parental/caregiver consent was excluded from the study and all gave verbal assent. Details about the economic or ethnic background of the children were not recorded.

#### Materials

##### Stimuli

Animal images and disgust and happy faces were identical to those in Experiment 1. In addition, 10 images (also 400 × 485 pixels) of fear faces (five men, five women), also from the NimStim Set of Facial Expressions (Tottenham et al., 2002), were used.

##### Fear and disgust beliefs questionnaires

The same measures of fear and disgust were used again in Experiment 2. Internal consistency for the FBQ was good before learning for the three animals; α = .75 for the cuscus subscale, α = .62 for the quokka subscale, α = .71 for the quoll subscale, and high after learning: .89, .85, and .86, respectively. For the DBQ, internal consistency was relatively low again before learning for all three animals; α = .49 for the cuscus subscale, α = .58 for the quokka subscale, α = .53 for the quoll subscale, and slightly higher after learning: .67, .58, and .52, respectively.

##### Disgust visual analogue scale (disgust VAS)

Children were asked how disgusting they thought each of the three animals was and responded in each case on a visual analogue-type scale. The scale consisted of a 100-mm continuous line from *not at all disgusting* to *extremely disgusting*, and children were instructed to mark the line according to how disgusting they felt the animal was. A disgust score between 0 and 100 was created for each animal. Evidence suggests that typically developing children’s cognitive abilities are sufficiently developed to understand and use a VAS by age 7 years; accuracy is less reliable in children under the age of 5 years (Shields, Palermo, Powers, Grewe, & Smith, 2003).

##### NRT

As in Experiment 1, the NRT was used to measure avoidance preferences. This experiment measured avoidance preferences toward all three animals: negative-paired (fear- or disgust-paired depending on group), happy-paired, and unpaired.

#### Procedure

Children completed the DBQ and disgust VAS by hand, followed by the computerized FBQ. Children in the disgust group received the same vicarious learning procedure as in Experiment 1: one animal with disgust faces, one with happy faces, and one unpaired. Children in the fear group saw fear faces instead of disgust faces as in previous studies using this procedure (e.g., Askew & Field, 2007); therefore, one animal was seen with fear faces, one was seen with happy faces, and one was unpaired. Children then completed the FBQ, DBQ, and disgust VAS for a second time, followed by the NRT. Finally, children were fully debriefed and given correct information and worksheets about the animals.

### Results

#### Fear beliefs

An initial four-way 2 (group: disgust vs. fear) × 3 (pairing type: negative, happy, unpaired) × 2 (time: before vs. after) × 3 (counterbalancing order: cuscus disgust-paired, quokka disgust-paired, and quoll disgust-paired) mixed ANOVA examined whether there were any effects of counterbalancing group on children’s fear beliefs. None were found and the analysis was subsequently collapsed into a three-way 2 (group: disgust vs. fear) × 3 (pairing type: negative, happy, unpaired) × 2 (time: before vs. after) mixed ANOVA. There was a significant main effect of pairing type, *F*(2, 112) = 9.40, *p* < .001, η_p_^2^ = .14, but not time or group. The Pairing Type × Group, *F*(2, 112) = 4.28, *p* = .016, η_p_^2^ = .07, and Pairing Type × Time, *F*(2, 112) = 18.96, *p* < .001, η_p_^2^ = .25, interactions were significant, but the Time × Group interaction was not.

**Figure 4 fig4:**
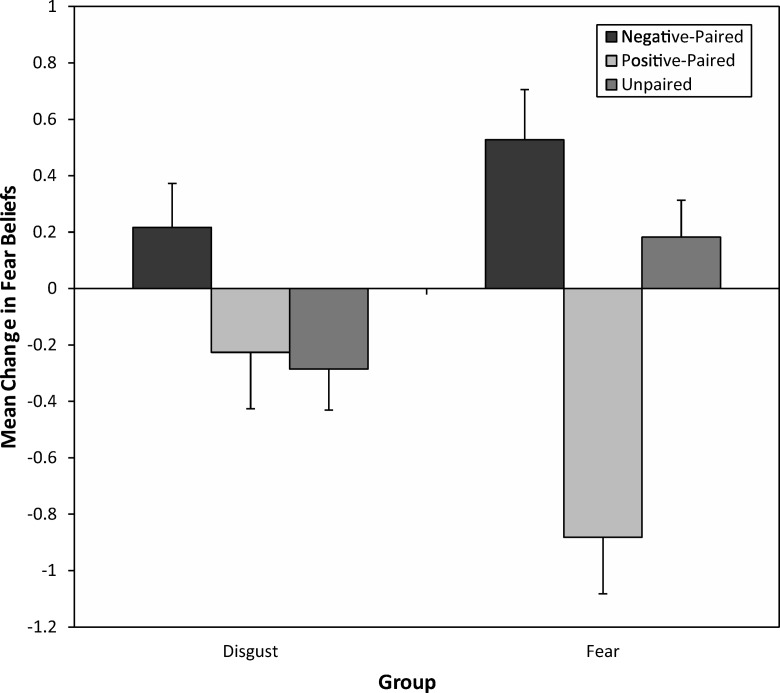
Mean (and *SE*) changes in fear beliefs following fear and disgust vicarious learning for negative-paired, happy-paired, and unpaired animals.

The Pairing Type × Time interaction, important for testing predictions, was significant, *F*(2, 112) = 18.96, *p* < .001, η_p_^2^ = .25, showing that fear beliefs changed because of vicarious learning, increasing or decreasing depending on the type of face animals had been seen with. Planned comparisons found that fear beliefs increased significantly for animals seen with negative (disgust and fear) faces, *F*(1, 56) = 9.25, *p* = .004, η_p_^2^ = .14, and decreased for animals seen with positive happy faces, *F*(1, 56) = 13.24, *p* = .001, η_p_^2^ = .19. In addition, the critical Pairing Type × Time × Group interaction was significant, *F*(2, 112) = 8.16, *p* < .001, η_p_^2^ = .12, indicating a significant difference in the vicarious learning effects between the disgust and fear vicarious learning groups. Thus, this interaction was broken down further in two two-way 3 (pairing type: negative, happy, unpaired) × 2 (time: before vs. after) repeated measures ANOVAs performed on each group. For children in the disgust group, there was a significant Pairing Type × Time interaction, *F*(2, 56) = 4.03, *p* = .023, η_p_^2^ = .13, showing that children’s fear beliefs for animals changed as a result of vicarious learning. Planned comparisons showed that fear beliefs increased for animals seen with disgust faces, *F*(1, 28) = 6.27, *p* = .018, *r* = .43 (medium-sized effect), compared with unpaired animals, but did not change for animals seen with happy faces, *F*(1, 28) = 0.11, *p* = .74, *r* = .06. In the fear group, the crucial Pairing Type × Time interaction was also significant, *F*(1.65, 46.20) = 20.24, *p* < .001, η_p_^2^ = .42 (Greenhouse–Geisser). Comparisons indicated that increases in children’s fear beliefs due to fear pairing were approaching borderline significance, *F*(1, 28) = 3.19, *p* = .085, *r* = .32, showing a medium-sized effect (Cohen, 1988, 1992), and decreases due to happy pairing were highly significant, *F*(1, 28) = 25.11, *p* < .001, *r* = .69. Figure 4 displays mean changes in fear beliefs following vicarious learning in the two groups.[Fig-anchor fig4]

#### Disgust beliefs

**Figure 5 fig5:**
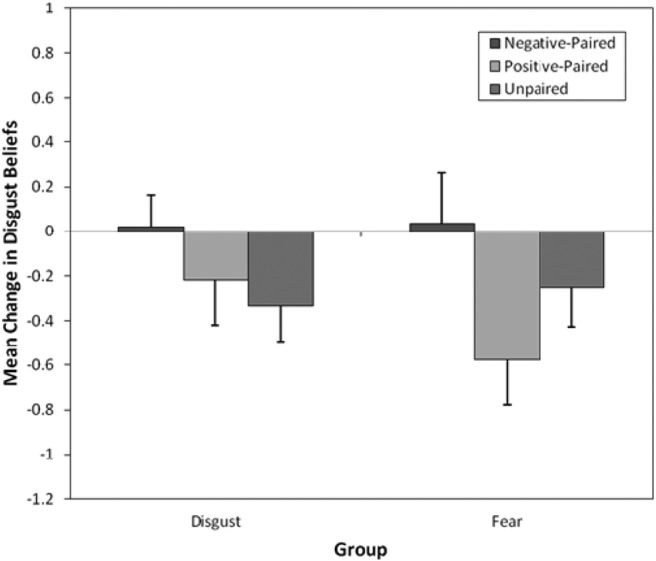
Mean (and *SE*) changes in disgust beliefs following fear and disgust vicarious learning for negative-paired, happy-paired, and unpaired animals.

Figure 5 displays mean changes in disgust beliefs for negative, happy, and unpaired animals following vicarious fear and disgust group learning. A preliminary four-way 2 (group: disgust vs. fear) × 3 (pairing type: negative, happy, unpaired) × 2 (time: before vs. after) × 3 (counterbalancing order: cuscus disgust-paired, quokka disgust-paired, and quoll disgust-paired) mixed ANOVA found no effect of counterbalancing order on children’s disgust beliefs. Thus, the analysis was collapsed into a three-way 2 (group: disgust vs. fear) × 3 (pairing type: negative, happy, unpaired) × 2 (time: before vs. after) mixed ANOVA. There was a significant main effect of time, *F*(1, 56) = 5.99, *p* = .018, η_p_^2^ = .10, but not pairing type or group. All interactions but one were nonsignificant, including the important Group × Pairing Type × Time interaction, which showed that vicarious learning was no different in the two groups. The other critical interaction, the Pairing Type × Time interaction, was significant, *F*(1.67, 93.24) = 4.13, *p* = .025, η_p_^2^ = .07 (Greenhouse–Geisser).[Fig-anchor fig5]

Planned contrasts found that disgust increased for disgust-paired and fear-paired animals (prelearning: *M* = 2.53, *SD* = 0.93; postlearning: *M* = 2.56, *SD* = 1.09) significantly more than unpaired animals (prelearning: *M* = 2.59, *SD* = 0.92; postlearning: *M* = 2.30, *SD* = 0.96), *F*(1, 56) = 7.24, *p* = .009, *r =* .34. Figure 4 shows that actual increases were slight, and differences were mainly due to decreases in disgust ratings for unpaired animals. But changes in disgust beliefs for happy-paired animals (prelearning: *M* = 2.55, *SD* = 0.83; postlearning: *M* = 2.16, *SD* = 1.01) were no different from those for unpaired animals, *F*(1, 56) = 0.45, *p* = .51, *r* = .09.

#### Disgust VAS

**Figure 6 fig6:**
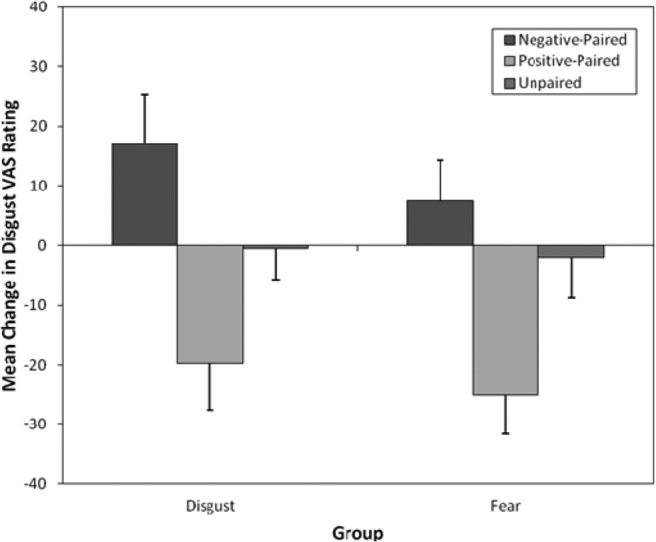
Mean (and *SE*) changes in self-reported disgust visual analogue scale (VAS) ratings following fear and disgust vicarious learning for negative-paired, happy-paired, and unpaired animals.

Figure 6 displays the mean change in disgust VAS ratings for the positive, negative, and unpaired animals following vicarious fear and disgust learning. Again, a four-way 2 (group: disgust vs. fear) × 3 (pairing type: negative, happy, unpaired) × 2 (time: before vs. after) × 3 (counterbalancing order: cuscus disgust-paired, quokka disgust-paired, and quoll disgust-paired) mixed ANOVA found no evidence that counterbalancing order affected children’s vicarious learning of disgust and counterbalancing order was removed from the main analysis.[Fig-anchor fig6]

A three-way 2 (group: disgust vs. fear) × 3 (pairing type: negative, happy, unpaired) × 2 (time: before vs. after) mixed ANOVA performed on changes in disgust VAS scores found a significant main effect of pairing type, *F*(2, 112) = 3.43, *p* = .036, η_p_^2^ *=* .058, but not of group or time. All interactions were nonsignificant except for the important Pairing Type × Time interaction, *F*(2, 112) = 14.44, *p* < .001, η_p_^2^ = .21, which indicated that vicarious learning had an effect on children’s disgust. However, there was no significant Group × Pairing Type × Time interaction, *F*(2, 112) = 0.19, *p* = .83, η_p_^2^ = .003; thus, effects of vicarious learning were similar in both groups.

The Pairing Type × Time effect was examined further by planned contrasts. Disgust ratings for the negative-paired (fear- and disgust-paired) animal (prelearning: *M* = 44.78, *SD* = 30.72; postlearning: *M* = 57.00, *SD* = 33.35) increased significantly more than those for the unpaired animal (prelearning: *M* = 41.53, *SD* = 31.88; postlearning: *M* = 40.29, *SD* = 30.84), *F*(1, 56) = 4.82, *p* = .032, *r =* .28. Similarly, disgust ratings for happy-paired animals (prelearning: *M* = 53.19, *SD* = 29.64; postlearning: *M* = 30.79, *SD* = 30.28) decreased more than those for unpaired animals, *F*(1, 56) = 10.09, *p* = .002, *r =* .39.

#### Avoidance preferences

**Figure 7 fig7:**
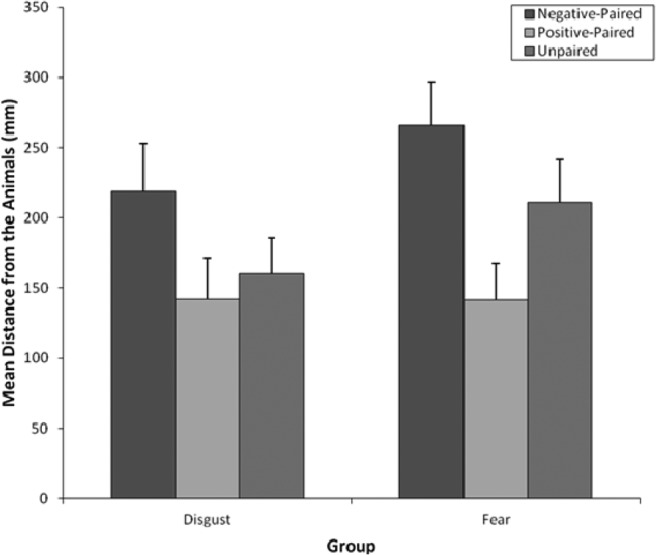
Mean (and *SE*) distances (mm) following fear and disgust vicarious learning for negative-paired, happy-paired, and unpaired animals in the nature reserve task.

Figure 7 shows mean distances from the negative (disgust and fear), happy, and unpaired animals for both vicarious learning groups in the NRT. A two-way 2 (group: disgust vs. fear) × 3 (pairing type: negative, positive, unpaired) mixed ANOVA was performed on avoidance preferences data. There was a significant (Greenhouse–Geisser adjusted) main effect of pairing type, *F*(1.79, 112) = 8.08, *p* = .001, η_p_^2^ = .13, and planned comparisons indicated greater avoidance of the negative-paired (fear- and disgust-paired) animal compared to the unpaired animal, *F*(1, 56) = 7.81, *p* = .007, *r* = .35, but there was no significant avoidance of the happy-paired animal compared to the unpaired animal, *F*(1, 56) = 2.57, *p* = .12, *r =* .21. The main effect of type of vicarious learning group, *F*(1, 56) = 1.14, *p* = .29, *r =* .14, and the Pairing Type × Group interaction, *F*(1.79, 100.28) = 0.66, *p* = .52, η_p_^2^ = .01, were not significant. Therefore, negative-paired animals were significantly avoided compared to control animals, regardless of whether fear or disgust faces were presented with animals during negative learning.[Fig-anchor fig7]

#### Relationship between fear, disgust, and avoidance

As in Experiment 1, correlational analyses were conducted on controlled scores: changes in scores for fear- and disgust-paired animals relative to unpaired animals. There were significant correlations between increases in fear beliefs and avoidance preferences in the fear, *r*(27) = .40, *p* = .033, and disgust, *r*(27) = .47, *p* = .01, groups. Increases in disgust beliefs did not correlate significantly with avoidance preferences for negatively paired animals in either group: fear, *r*(27) = .22, *p* = .26; disgust, *r*(27) = .09, *p* = .64. However, increases in disgust VAS scores were significantly associated with avoidance preferences for negatively paired animals in both the fear, *r*(27) = .45, *p* = .014, and disgust, *r*(27) = .39, *p* = .039, groups. Finally, increases in fear beliefs showed a borderline significant correlation with disgust VAS scores in the fear group, *r*(27) = .36, *p* = .059, but not the disgust group, *r*(27) = .16, *p* = .41.

**Figure 8 fig8:**
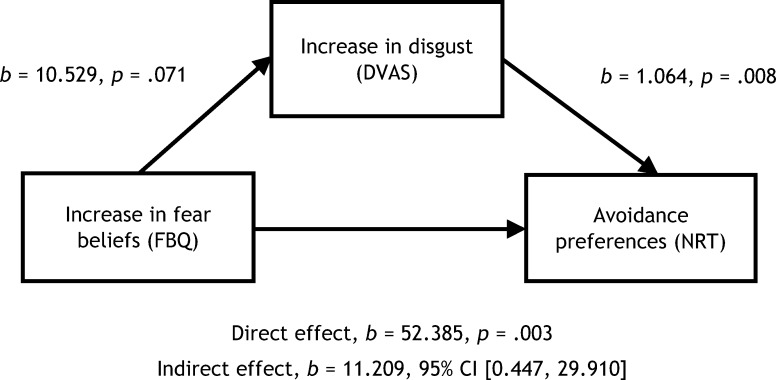
Mediation model of increases in children’s fear beliefs following negative (disgust and fear) vicarious learning as a predictor of avoidance preferences and mediated by disgust beliefs for the animals. DVAS = disgust visual analogue scale; FBQ = fear beliefs questionnaire; NRT = nature reserve task; CI = confidence interval.

A Hayes (2013) mediation analysis showed a significant indirect effect of increases in fear beliefs on avoidance preferences for animals that was mediated by disgust VAS scores, *b* = 11.209, BCa CI [0.157, 28.445], showing a small effect, *k*^2^ = .083, BCa CI [.005, .206]. Figure 8 shows the mediation model (note that the relationship between changes in fear beliefs and changes in disgust VAS scores was only borderline significant, *p* = .071). Mediation analyses conducted on disgust and fear groups separately did not reach significance.[Fig-anchor fig8]

## General Discussion

Two experiments investigated the effects of disgust and fear vicarious learning on children’s fear beliefs, disgust beliefs and ratings, and avoidance preferences for novel animals. Results indicated that (a) disgust vicarious learning led to increases in children’s fear beliefs for animals in both experiments, (b) children rated animals in Experiment 2 as more disgusting after seeing them in both fear- and disgust-paired vicarious learning, (c) children in both experiments preferred to avoid animals they had seen in disgust or fear vicarious learning procedures compared to control animals, (d) increases in fear beliefs for animals were associated with increases in disgust beliefs due to fear and disgust vicarious learning, (e) increases in disgust beliefs (Experiment 1) and disgust ratings (Experiment 2) for fear- and disgust-paired animals were associated with increases in avoidance preferences for these animals relative to control animals, and (f) the relationship between increases in fear beliefs and avoidance preferences was mediated by increases in disgust for animals.

Both studies showed that children’s fear beliefs and avoidance preferences for animals increased after they saw them together with adult faces expressing disgust. Moreover, increases in children’s fear beliefs for disgust-paired animals were related to their disgust and avoidance preferences for them: Increased fear beliefs for animals were associated with a greater preference to avoid them and this was mediated by increases in disgust for the animals. Matchett and Davey (1991) suggested in their disease-avoidance model that one way the disgust emotion may play a role in fears is by increasing avoidance aimed at preventing transmission of contamination and disease. Consequently, a stimulus that becomes associated for whatever reason with disgust might subsequently be feared and avoided. Rozin and Fallon (1987) proposed that disgust might be acquired via the nonverbal expressions of others. The current findings support these suggestions, showing that associating an animal with disgust during vicarious learning, by observing an adult expressing disgust for it, increases children’s fear beliefs for the animal and leads to a disgust-mediated preference to avoid it.

One implication of the disease-avoidance model is that we would also expect to find increases in how disgusting children find the animals. Experiment 1 did not find direct evidence of this: A significant overall effect of vicarious learning on disgust beliefs appeared to be due in the main to the difference in changes for fear- and happy-paired animals. Similarly, significant effects of negative vicarious learning on disgust beliefs in Experiment 2 appeared to be mainly due to decreases in disgust beliefs for unpaired control animals, with disgust beliefs for negatively paired animals increasing only slightly. Nevertheless, despite this lack of significant overall increases in disgust beliefs, correlational analyses for both experiments showed that the relationship between increases in fear beliefs and avoidance preferences was mediated by increases in children’s disgust for the animals. No difference was found between disgust and fear vicarious learning. One explanation for the findings may be that the measure of disgust beliefs was not sensitive enough to detect significant overall changes in disgust beliefs, despite these increases being related to fear belief measures. Indeed, one potential weakness of the disgust belief measure was that it was unclear whether unsatisfactory Cronbach’s alpha scores were due to low reliability of the scale or merely the low number of items; there were indications that the latter interpretation was most likely. Fortunately, a much clearer set of results was found using the disgust VAS scale in Experiment 2, which unambiguously indicated that both fear and disgust vicarious learning led to significant increases in how disgusting children rated the animals. Again, these increases in disgust were also significantly associated with avoidance preferences following disgust and fear vicarious learning, and there was a borderline significant (*p* = .059) association with increases in fear beliefs following fear vicarious learning. Thus, the disgust VAS may be a more sensitive measure of changes in children’s disgust for the animals, presumably due to responses being on a continuum rather than a discrete scale. A useful addition to future studies may be to also use a comparable fear VAS. This may allow greater precision in comparisons between fear and disgust learning.

One unexpected finding from Experiment 2 was that fear beliefs increased significantly for disgust-paired but not fear-paired animals. This result is difficult to explain because previous studies have consistently demonstrated that fear vicarious learning leads to significant increases in children’s fear beliefs (Askew & Field, 2007; Askew et al., 2008; Dunne & Askew, 2013), heart rate (Reynolds, Field, & Askew, 2014), avoidance preferences (Askew et al., 2013; Dunne & Askew, 2013), and avoidance behavior (Askew & Field., 2007; Reynolds et al., 2014). Moreover, Experiment 2 found that avoidance preferences were greater for fear-paired animals compared with controls, suggesting that a change in threat-related cognitions had occurred. There was a small to medium effect (*r* = .23) of increased fear beliefs that approached but did not reach conventional levels of significance (*p* = .085), and the most likely explanation is that the sample size in this group (*N* = 29) was too small to provide sufficient power to detect this effect. Some indirect support for this comes from the mediation analysis, which indicated that increases in fear beliefs for animals following disgust and fear vicarious learning were related to increases in avoidance preferences and were mediated by increases in disgust beliefs. Overall, the significant difference between fear and disgust groups was most likely due to differences in sizes of effects of happy pairing, which was much larger in the fear group (*r* = .59) than the disgust group (*r* = .04). Given that the happy vicarious learning procedure was identical in the two groups, it is strange that it produced much larger effects in the fear group. The difference in effects was in part down to fear beliefs increasing for unpaired animals in the fear group (perhaps owing to greater generalization from fear-paired learning to unpaired animals), but it was not only due to this. The large difference in effects is difficult to explain, but it seems that seeing fear as opposed to disgust faces with some animals also affects decreases in children’s fear beliefs for animals seen with happy faces.

In their influential article on disgust, Rozin and Fallon (1987) suggested that disgust is transmitted from person to person, for example, from adult caregivers to children via social referencing. The current study demonstrates that disgust can be transmitted from adult stranger to child. An interesting follow-up study, with important developmental implications, would be to investigate whether children can also vicariously learn disgust from similarly aged peers. The mechanisms underlying vicarious disgust learning also remain unspecified. Vicarious fear learning is generally assumed to be a form of associative learning (e.g., Askew & Field, 2007, 2008; Bandura, 1969; Berger, 1962; Hygge, 1976; Mineka & Cook, 1993) in which a model’s fearful response to an animal or object CS acts as a US for observing individuals, who associate this US with the CS as a result of their contiguous presence (Askew & Field, 2007, 2008; Mineka & Cook, 1993). Even if we accept that disgust vicarious learning is also associative, the specific associations underlying vicarious disgust learning and vicarious fear learning could theoretically be very different from each other. For example, disgust-related events might lead to the formation of associations more akin to those found in evaluative conditioning, which may be qualitatively different from those underlying classical conditioning (see, e.g., Hofmann, De Houwer, Perugini, Baeyens, & Crombez, 2010). This would be important because this type of learning may be less affected by contingency awareness (e.g., Baeyens, Eelen, & Van den Bergh, 1990) and extinction procedures (Baeyens, Crombez, Van den Bergh, & Eelen, 1988), although the evidence for this is not entirely unequivocal (Hofmann et al., 2010). Future studies could investigate the mechanisms underpinning disgust vicarious learning by testing the need for awareness of pairing contingencies and whether learned responses to animals extinguish during animal-only trials. Furthermore, it would be possible to determine whether CS–US associations underpin disgust vicarious learning using a US revaluation procedure.

The vicarious learning procedure used in the experiments represents a harmless laboratory analogue of how children acquire fears in the real world. As a prospective paradigm with nonfearful children, it can potentially inform theory about fear acquisition and support the development and assessment of new fear prevention and intervention strategies. For obvious ethical reasons, the mild nature of the manipulation used meant that changes in fear responses were small compared with phobic levels of fear. But they are significant for several reasons. First, a vicarious learning episode in the real world is likely to be more intense than in the current study, involving real animals and people and richer information (e.g., sound and movement) that could lead to greater learning. The current findings show that even relatively unthreatening learning experiences can create avoidance preferences, which in turn could mean that a stimulus is avoided and beliefs about the threat it poses are not disconfirmed. Moreover, previous studies looking at fear vicarious learning have shown that similar increases in fear beliefs and avoidance preferences are related to actual behavioral avoidance and increased heart rate when children actually encounter the stimuli (Reynolds et al., 2014).

Given that CS–US associations are generally believed to be formed during vicarious learning (Askew & Field, 2007, 2008; Mineka & Cook, 1993), there are likely to be other implications for the formation of fears. Even relatively innocuous CS–US associations, once learned, can potentially become pathological at a later date because of US revaluation processes (Rescorla, 1974; White & Davey, 1989). If the US is revalued as more aversive than first thought, then the response to the CS will also increase if CS–US associations underpin learning, although initial attempts by Askew et al. (2008) to inflate the aversiveness of a US following vicarious learning were unsuccessful at affecting responses to the CS. An additional means by which existing fear beliefs can influence the development of more serious fear is via the creation of negative outcome expectancies that impact future learning (Davey, 1997; Field & Davey, 2001). Expectancies about the outcome (US) of an encounter with a CS can enhance what is learned about it in a subsequent CS–US learning event: Entering an aversive learning event with existing expectancies that the outcome (US) will be negative can enhance fear learning for the CS. Thus, disgust-related vicarious learning about a CS may prepare children to learn fear should they encounter the CS in a threatening or harmful situation in the future. Finally, the current findings have implications for prevention and intervention in fear acquisition. Increased understanding about the role of observational disgust learning in fears can inform caregivers and those working with children about the effect of others’ behavior on children’s fear-related cognitions for stimuli. In addition, knowledge about the role of disgust in the formation of avoidance preferences during vicarious learning events can give clinicians and theorists greater insight into the processes involved in some fears. Understanding that fear and disgust can develop via CS–US associations, if this is indeed the case, could potentially allow clinicians to make better predictions about the acquisition, course, and treatment of specific fears and phobias.

A potential limitation of the study was that disgust sensitivity was not measured. Given the importance of disgust sensitivity and propensity in some fears (e.g., van Overveld et al., 2006), it would be interesting to investigate whether these characteristics affect susceptibility to disgust vicarious learning. One possibility, for example, is that the relationship between changes in disgust beliefs and changes in fear beliefs is mediated by disgust sensitivity. It is also worth noting that statistical power to detect vicarious learning effects is reliant on the discriminability of the stimuli used. If children are confused about the identity of animals, this could potentially decrease within-child and between-groups learning effects. Thus, learning for paired animals compared with unpaired animals and, more important here, differences in learning between disgust and fear groups could potentially be underestimated if there is confusion about the visual appearance of animals. The discriminability of animals was not directly examined here, but these animals have been used successfully in previous vicarious learning studies (e.g., Askew & Field, 2007; Askew et al., 2008, 2013; Dunne & Askew, 2013; Reynolds et al., 2014).

To summarize, two experiments showed that disgust vicarious learning can lead to increases in children’s fear beliefs and avoidance preferences for animals, and these increases are related to increases in how disgusting they feel the animals to be following learning. Fear vicarious learning also led to increases in children’s disgust ratings for animals, suggesting a bidirectional relationship between disgust and fear in vicarious learning.
