# Correction to Askew et al. (2014)

**DOI:** 10.1037/abn0000008

**Published:** 2014-09-22

The article “The Effect of Disgust and Fear Modeling on Children’s Disgust and Fear for Animals” by Chris Askew, Kübra Çakır, Liine Põldsam, and Gemma Reynolds (*Journal of Abnormal Psychology*, 2014, Vol. 123, No. 3, pp. 566–577. http://dx.doi.org/10.1037/a0037228) was missing the acknowledgement of the funding source in the author note. The work in this article was supported by the Economic and Social Research Council: ERSC (grant number ES/J00751X/1). The online version of this article has been corrected.

